# Evaluation of the Olfactory Quality of Roasted Coffee Beans Using a Digital Nose

**DOI:** 10.3390/s22228654

**Published:** 2022-11-09

**Authors:** Juan Diego Barea-Ramos, Gema Cascos, Marta Mesías, Jesús Lozano, Daniel Martín-Vertedor

**Affiliations:** 1 Technological Institute of Food and Agriculture (CICYTEX-INTAEX), Junta of Extremadura, Avda. Adolfo Suárez s/n, 06007 Badajoz, Spain; 2 Institute of Food Science, Technology and Nutrition (ICTAN-CSIC), Jose Antonio Novais 10, 28040 Madrid, Spain; 3 Industrial Engineering School, University of Extremadura, Avda. de Elvas s/n, 06006 Badajoz, Spain

**Keywords:** roasted coffee beans, coffee quality, sensory analysis, volatile compounds, electronic nose

## Abstract

The roasting process is one of the critical points to obtain a product of the highest quality with certain sensorial properties including the aroma of coffee. Samples of coffee beans were roasted at different thermal treatment intensities with the aim of obtaining aromatic compounds detected with an electronic device. Sensory analysis, volatile compound profiling, and electronic nose analysis were carried out. Through principal component analysis (95.8% of the total variance of the data was explained by PC1 and PC2) and partial least squares discriminant analysis (the sum of the diagonal elements gave a hit rate of 94%), it could be demonstrated that the E-nose is able to discriminate roasted coffee beans subjected to different thermal treatments. Aromatic profiling was carried out by a testing panel and volatile compounds (VOCs) for the discrimination of roasted coffee samples. Alcohols, aromatics, esters, ketones and furanone were found in higher proportions in samples at the lowest thermal treatment. The VOCs with positive attributes were 1-(4-nitrophenyl)-3-phenylamino-propenone, carboxylic acids, 2-methoxy-4-vinylphenol, and 2-phenylethyl alcohol, while the compounds with negative ones were 2-methyl-furan, 2,5-dimethyl-pyridine, 2-methyl-butanal, and 2-furfurylthiol. The PLS model allows for the quantification of the positive and negative aromas ($R_{CV}^{2}$ = 0.92) of roasted coffee by using the E-nose. Therefore, the E-nose, that is, an inexpensive and nondestructive instrument, could be a chemometric tool able to discriminate between different qualities of coffee during processing.

## 1. Introduction

Coffee is the most popular drink in the world after water due to its unique taste, aroma, and relaxing or aphrodisiac effects [1]. This special flavor is achieved through the roasting of coffee beans, which is a complex process that involves mechanical, thermal, and chemical changes in the product. The final quality of the coffee beverage and the price of the product is affected by the roasting process [2]. Roasted coffee is the world’s 261st most traded product, with a total trade of $11 B [3]. Thus, the largest exporter of roasted coffee is Switzerland ($2.49 B), while the largest importer of this product is France ($1.3 B) [3].

The most important genus of the botanical coffee family is *Coffea*, with more than 25 species, native from Africa to the Indian Ocean islands. Arabica and Robusta are the main varieties harvested in the world. The plants (*Coffea canephora* and *Coffea arabica*) are grown in Southeast Asia, India, America, and Africa at 800 m in altitude, where *C. arabica* is more robust than *C. canephora* [4].

Roasted coffee processing is divided into three different phases: (i) drying: green coffee beans are dried by vaporization processes; (ii) pyrolysis: coffee is submitted to thermal treatment between 160–200 °C for 4–12 min, when Maillard reactions occur [5] and volatile organic compounds (VOCs) and semi-volatile organic compounds (SVOCs) are produced; (iii) cooling: the coffee beans are cooled rapidly by exposure to ventilation or cold water [2].

Regarding the sensory properties of the two main coffee species, Arabica coffee is known for its aroma, acidity, and for having less body than Robusta coffee [6,7]. In general, Robusta coffee is characterized by a bitter taste and astringency, while Arabica develops a better flavor, and it is more intense in aroma [8]. Nevertheless, the physical and organoleptic quality of these species are influenced by climatic conditions, soil type, crop management, postharvest treatment, and insect damage [9].

The aroma of coffee is a very relevant aspect and for years, has motivated researchers in the investigation of the main chemicals produced after the roasting process [10]. Green coffee contains thousands of chemicals that are responsible for the taste and aroma of the final product. The most prominent sensory attributes after roasting are bitter, astringent, burnt, liquor, tobacco, toasted, nutty, almond, and buttery. These aromas depend on the type and degree of roasting applied to the coffee beans [11,12,13]. Roasted coffee contains a different chemical composition than green coffee, such as sucrose, free amino acids, chlorogenic acids, trigonelline, and the breakdown of polysaccharides and proteins [14]. Thus, more than 900 volatile compounds were identified in roasted coffee. The most characteristic belonging to the families of furans, pyrazines, ketones, pyrroles, phenols, hydrocarbons, acids and anhydrides, aldehydes, esters, alcohols, sulfur compounds, and others [15]. For the analysis of volatile compounds in roasted coffee, SPME-GC-MS was used [16]. This technique is widely used because of its high resolution, sensitivity, and short analysis times. It performs the separation of volatile and semi-volatile compounds at temperatures up to 350–400 °C. By means of a calibration curve of the corresponding standards, the number of individual components present in a sample can be determined by gas chromatography. Some of them are universal, while others are more selective and respond only to some of the components in a mixture [17].

Sensory evaluation of coffee beans allows for the collection, evaluation, and analyzation of the properties of the food [18] that require experts in sensory analysis. In the coffee industry, the cupping technique involves tasting three to ten cups of the same coffee prepared according to conditions of temperature, contact time, water/coffee ratio, water quality, and brewing method [12]. The attributes of the coffee are then scored from 0 to 10, with the highest quality being a balanced intensity of acidity and aroma and good aroma and flavor with a medium body [19]. The aspects mentioned above contrast with the sensory classification of the coffee quality at the industry level that is carried out by industry staff [12].

Having electronic equipment to determine and classify aromas in the agrifood industry is of great interest. Different aromas in different agrifood or in the oliviculture sector such as in edible oils can be detected by a small electronic device, the E-nose [20,21,22]. This device could assist in gas chromatography and sensory evaluation because it is a powerful, useful, cheap, and fast instrument which can be used to examine a wide range of samples. The E-nose system makes it possible to contribute to the characterization of the aroma properties of the original coffee, allowing for the detection of thresholds suitable for evaluating the quality of the coffee and also offering a certain standardization in the tasting process, being very useful at an industrial level for quality control, safety, and traceability [23]. Therefore, the general objective of this work is to demonstrate that a data matrix obtained by the E-nose allows for the correlation of the signals acquired from the sensor array with the organoleptic and volatile organic compound characteristics of different roasted coffee samples. The differentiation of the qualities of samples will also allow the E-nose to be used as a chemometric measurement tool for the first classification of roasted coffee.

## 2. Materials and Methods

### 2.1. Samples

**Figure 1:**
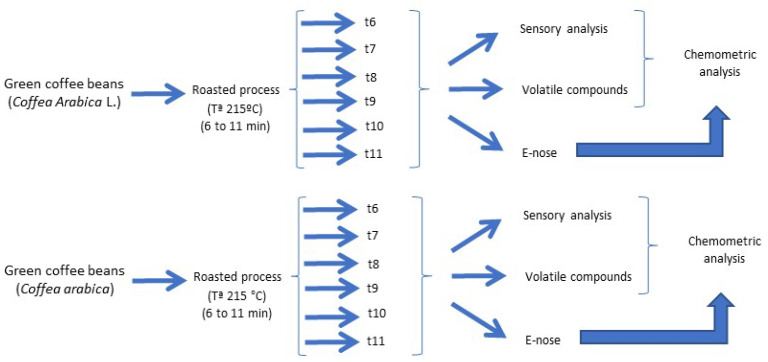
Diagram of the experiment.

Samples of green coffee beans (2 kg) from *Coffea arabica* L. were purchased in a local factory during the 2021/22 crop season in the autonomous community of Madrid (Spain). Samples were uniformly distributed in porcelain plates forming a single layer. Green coffee beans were roasted from low to high roasts (6–11 min) at 215 °C using a conventional oven (model 210, J.P. Selecta^®^, Barcelona, Spain). Each treatment was repeated three times. The temperature of the oven was verified with two coupled Pt100 temperature sensors (Autoclave Load RTD, Type 69A, United Kingdom). The sensors were put in the center of the container where the sample was placed and were connected to a computer, allowing the temperature profile inside the oven during the processing time to be recorded, ensuring that the desired heat penetration was attained [24,25]. Samples were held at room temperature for 24 h, and after that, they were immediately analyzed. A diagram of the experiment is shown in Figure 1.

### 2.2. E-Nose Measurements

An inexpensive and accurate electronic device, the E-nose, was used to analyze the roasted coffee bean samples. This miniaturized piece of equipment was developed by the Industrial Engineering School of the University of Extremadura. The instrument is composed of 4 digital gas sensors that generate 11 different signals, described in Table 1. These sensors belong to the latest generation of gas sensors that incorporate within the same housing one or more gas sensors and other sensors (temperature, humidity, atmospheric pressure), together with the signal processor, the controller of the whole system, and the communication module via I2C. These types of sensors are subject to numerous changes in the market, so at the time of reading this article, some sensors may not be available, although there will probably be similar ones. In this sense, the instrumentation system is prepared to connect to most of these sensors. In this sense, two separate I2C interfaces have been used due to the different supply voltages (+1.8 V and +3.3 V) of the sensors. On the other hand, some of these sensors include intelligent algorithms to process the raw signals to output TVOC (total volatile organic compounds) and equivalent CO_2_ (_e_CO_2_) prediction values. Additionally, SGP30 provides raw signals for H_2_ and ethanol. Most of the sensors in use are discontinued. The values of multiple sensors are all calculated from the same sensor resistance using an algorithm.

The instrumentation system is controlled by a 32-bit microcontroller, (PIC32MM0256GPM048 from Microchip), which performs the main operations: sensor control and measurements; communications with smart devices by using a Bluetooth low-energy RN4871 tiny module from Microchip; and wired UART communications with other devices. More details on the electronic design can be found in [26]

**Figure 2:**
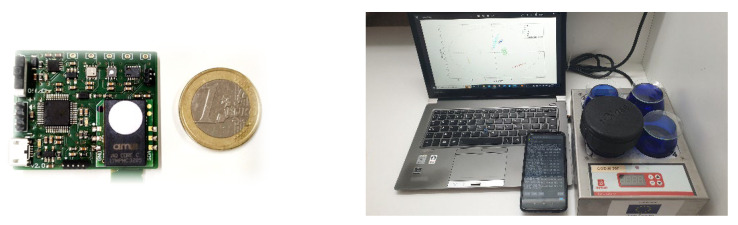
Electronic nose device (**left**) and the components involved in E-nose measurements (**right**).

The measurement procedure with the E-nose was carried out at the same time as the tasting panel. Figure 2 shows the components of the analysis with the electronic device. Three g of roasted coffee beans were added to standard cups, covered with a watch glass, and left at room temperature. A cup without sample was used as a reference. First, the watch glass was removed from the cup with the sample and the E-nose was placed over the cup for 60 s while the sensor signals were recorded every second. Then, desorption was performed by placing the E-nose on the cup without a sample for 30 s to bring the gas sensor signal back to the baseline. Each sample was analyzed six times. All measurements were conducted one after another because there may have been errors in the E-nose measurements which would cause deviations in the results. Finally, all measurements were sent to an external smart electronic device via Bluetooth. After that, data were sent to the computer, and chemometric analysis was performed.

### 2.3. Sensory Analysis

The sensory analysis was conducted in a tasting room in the research center CICYTEX (Extremadura, Spain) by eight trained tasters. Three g of roasted coffee beans was introduced in a standard cup, covered with a watch glass, and placed in a heating block at 25 °C. Each sample was classified according to the intensity of the coffee odor. The positive and negative attributes perceived by the tasters were evaluated on a scale of 0 to 10 points. The test was considered valid when the coefficient of variation was less than 20%.

### 2.4. Analysis of Volatile Compounds

VOCs in roasted coffee beans were analyzed using gas chromatography. Two grams of sample was placed in a vial with an NaCl solution (30% *w*/*v*). Then, the 65 mm polydimethylsilane/divinylbenzene (PDMS/DVD) StableFlex fiber (Supelco) absorbed the headspace of the samples for 30 min at 40 °C. To carry out the desorption of the compounds, the fiber was introduced into the Agilent DB WAXetr GC-MS with a capillary column (60 m × 0.25 mm × 0.25 mm) at 250 °C for 15 min. All volatile compounds were identified using the standard NIST 2.0 MS library. Results are presented in percentages of the area of each identified compound.

### 2.5. Multivariate Data Analysis

To characterize the sensor response curves of the data obtained by the E-nose, results were firstly processed by applying the formula of the maximum signal value minus the minimum signal value multiplied by 100 and subtracted by one: ((MAX − MIN) × 100 − 1). After that, principal component analysis (PCA) was carried out [27]. Next, partial least squares discriminant analysis (PLS-DA) was performed to identify the components or latent variables (LV) by discriminating samples of different qualities [28]. A confusion matrix was constructed to deduce cross-validation predictions. Further, the partial least squares regression (PLSR) method was used to build quantification models for the evaluation of coffee aromas perceived by the panelists and the negative ones. The chemometric analysis was described by Sánchez et al. [29]. Roasted coffee bean samples were divided into a calibration set that contained 70% of all the samples, and a validation one that contained all the remaining samples (30%). Samples were divided randomly between the two sets. Data analysis was performed using Matlab version R2016b, version 9.1 (The Mathworks Inc., Natick, MA, USA) with PLS_Toolbox 8.2.1 (Eigenvector Research Inc., Wenatchee, WA, USA).

### 2.6. Statistical Analysis

Significant differences and homogeneous groups of the descriptive sensory analysis were established by analysis of variance (ANOVA). When the difference between the mean values was significant (*p* < 0.05), a test of comparison of means was performed using the Tukey method (univariate analysis). Mean values and standard deviations are reported. The SPSS 18.0 software was used for statistical analysis (SPSS Inc., Chicago, IL, USA).

## 3. Results and Discussion

### 3.1. Roasted Coffee Beans Discriminated by the E-Nose

**Figure 3:**
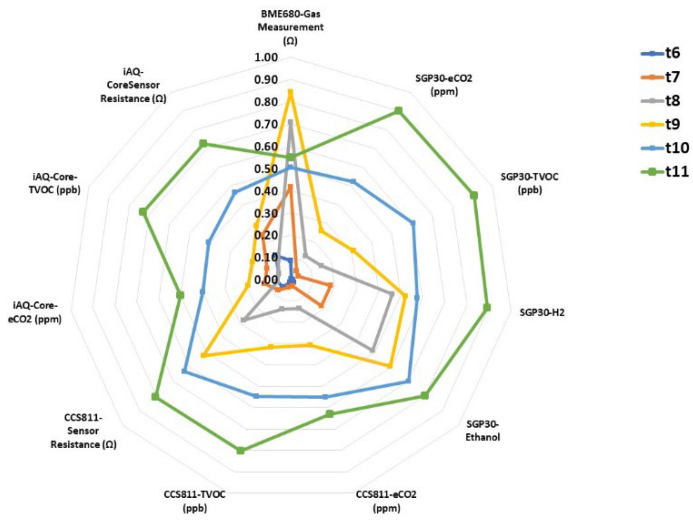
Radial plots of the sensors’ responses to roasted coffee beans submitted to different thermal treatments (t6–t11).

The classification of roasted coffee beans was conducted after performing a chemometric analysis of the results obtained with the sensors of the E-nose. The procedure to analyze both the volatile compounds with the E-nose and the sensory analysis was similar. Coffee samples were introduced in the standardized tasting cups and measurements of the headspace of the samples were performed with the electronic equipment. Figure 3 shows the electrical signal emitted by each sensor in the presence of the sample. A radial plot was drawn to figure out the different responses of the sensors to the olfactory pattern of the sample. Data from the E-nose was previously normalized according to the formula (X_i_ − X_MIN_)/(X_MAX_ − X_MIN_), where X_i_ is the experimental value measured for sample i; X_MIN_ is the minimum experimental value of the data series; and X_MAX_ is the maximum experimental value of the data series. Thus, the normalized values of the data series for each of the roasted coffee beans submitted to different thermal treatments were averaged, and each treatment was represented in the radial graph. It can be clearly seen that the amplitude of the signals from samples submitted to higher thermal treatments (t10 and t11) was generally of greater magnitude than the samples with a lower cooking intensity (t6 and t7). In addition, each curve represents clear differences, which could be due to the different volatile profiles among the samples. All the signals obtained by the different sensors responded with clear differences between the samples except for the BME680 Gas Measurement device whose response occurred with less intensity for the aromas of the different treatments.

**Figure 4:**
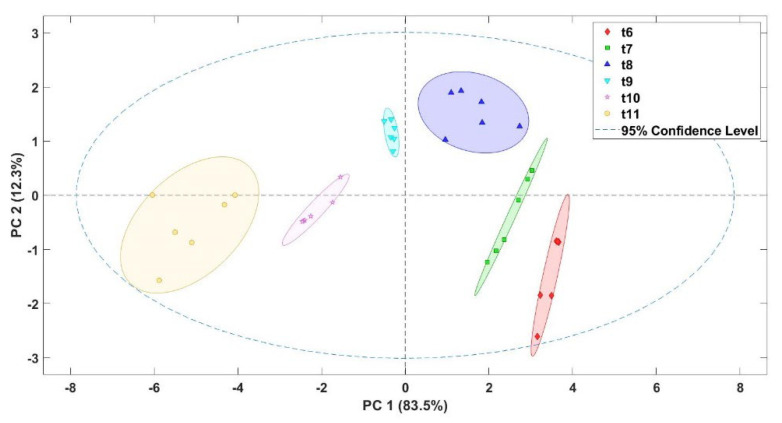
Score plot of the principal component analysis (PCA) for roasted coffee beans submitted to different thermal treatments (t6–t11).

The results obtained by the electronic nose were used to evaluate if the coffee samples subjected to different thermal roasting treatments were discriminated. Thus, principal component analysis (PCA) was performed to reduce data from the E-nose sensors to principal components that were graphically drawn according to the thermal treatments applied (Figure 4). Results showed that the E-nose was able to differentiate roasted coffee beans subjected to different thermal treatments. A value of 83.5% of the total variance of the data was explained by PC1, while PC2 explained 12.3%. The different classification groups of the samples moved from left to right in the different quadrants represented. This classification indicates that the roasted coffee samples had different characteristic aromatic profiles that allowed the sensors of the E-nose to react to the aromas of each sample.

A partial least squares discriminant analysis (PLS-DA) was also performed using leave-one-out cross-validation (Table 2). The confusion matrix of the model indicates that the sum of the diagonal elements gave a hit rate of 94%. Six samples from each class were used to construct the model, which allows for the discrimination of roasted coffee beans subjected to different thermal treatments according to their aromatic characteristics. Samples with intermediate thermal treatments were the ones that had a slightly higher percentage of error than the rest of the treatments, successfully predicted with an accuracy of 100%. Therefore, the model established could predict the correct discriminations of the roasted coffee beans submitted to different treatments.

The classification of coffee in different categories by using electronic devices has been previously described [30]. Rodríguez et al. [31] used this chemometric tool to classify different qualities of Colombian roasted coffee, searching for a product with a high commercial quality. Brudzewski et al. [32] discriminated coffee brands with different qualities, low, medium, and high-quality coffee types, by applying an electronic nose, obtaining results similar to those observed in the present study. Similarly, Gonzalez Viejo et al. [33] estimated the intensity and aroma of roasted coffee using a low-cost and portable electronic nose and machine learning modelling.

### 3.2. Aromatic Profile of Roasted Coffee Beans

To verify the discrimination of the roasted coffee samples, the aromatic profile was evaluated. First, a descriptive sensory evaluation of roasted coffee beans was analyzed by a tasting panel to know the olfactory sensations of the coffee after being subjected to different roasting thermal treatments (Table 3). The tasting panel described whether the different samples had pleasant or unpleasant attributes that could discriminate roasted coffee beans based on their sensory quality. The positive attributes were related to the coffee aroma, while the negative ones were related to roasted or burned aromas. It should be noted that the positive aroma of coffee increased with the longest time of thermal treatment, until t9. From this treatment, the aromatic intensity progressively decreased until t11. The maximum aromatic intensity of the coffee was 5.7 points, and the minimum was 1.1. Regarding the negative attributes, roasted was the most significant attribute assessed by the panelists. In this case, samples submitted to the longest thermal treatments showed higher values for this attribute. It should be taken into account that the roasted odor is a descriptor related to burnt food associated with smoke from burning wood [6]. Coffee with this sensory defect was not detected by the panelists until the t9 treatment. From here, the negative aromatic intensity increased progressively until t11. The maximum defect intensity of the coffee detected by the panelists was 4.4 points, and the minimum was 2.1.

The sensory descriptive methods used were effective tools in identifying the sensory characteristics in the roasted coffee beans tested. Toledo et al. [15] evaluated different sensory aspects related to the quality of coffee beans such as cultivars, geographic origins, processing, roasting, and storage. However, the information gained from this research can be used to classify this product in the industry into different categories. Therefore, these results indicate that the final product obtained could be marketed for high or premium quality coffee. In the market, we can find a wide variation in coffee quality from different countries. Thus, the results obtained will help to classify coffee regarding its quality. It could be admitted that the treatment with a medium thermal intensity (t9) presented a greater aromatic intensity with a slight burning defect. In this sense, Zhang et al. [34] showed that a higher drying process in Robusta coffee presented greater defects in the samples, whereas Toledo et al. [15] reported that sensory defects in coffee seeds are caused by excessive microbial fermentation of the seeds during post-harvest processing.

**Figure 5:**
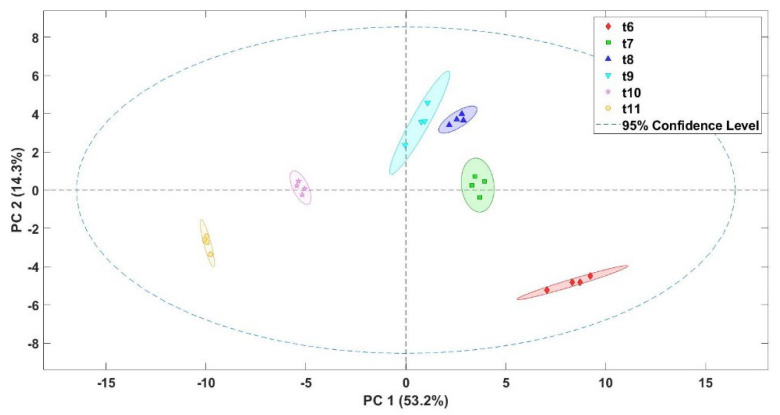
Score plot of the principal component analysis (PCA) for the VOCs identified in the roasted coffee beans submitted to different thermal treatments (t6–t11).

The principal component analysis of the VOCs from the data obtained by the E-nose was also assessed to figure out the possible differences between the roasted coffee bean samples (Figure 5). The first two principal components accounted for 67.5% of the variations seen in the data. As can be observed, the analysis allowed for the classification of samples in different groups. The beans with the highest thermal treatments (t10–t11) were in the negative zone of the PC1, and in contrast, the other treatments were in the positive zone. From these results, it could be admitted that the aromas of the samples were completely different from each other.

**Figure 6:**
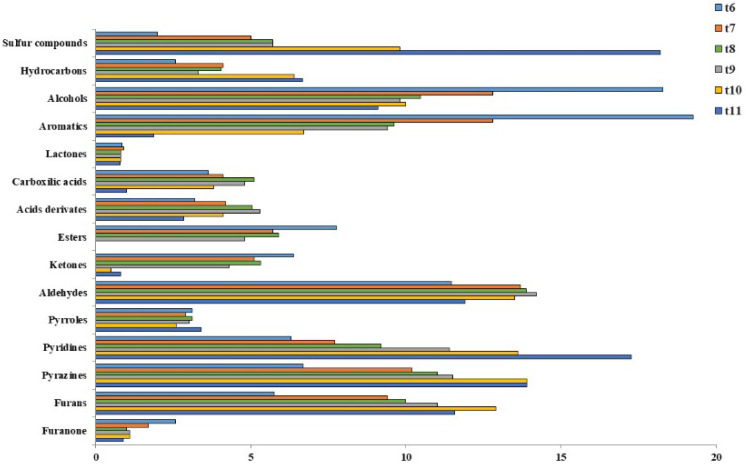
Chemical distribution of the VOCs identified in the roasted coffee beans submitted to different thermal treatments (t6–t11).

The VOCs of roasted coffee beans were classified into different types of chemical groups (Figure 6). The groups of VOCs with the highest representations in the samples were aromatics, alcohols, and sulfur compounds, followed by pyridines, aldehydes, and pyrazines. Some groups increased with the time of thermal treatment, probably due to the fact that the formation of certain VOCs is related to the negative aroma. The VOC groups with the highest increases were sulfur compounds, hydrocarbons, pyridines, and pyrazines [15,35]. On the contrary, alcohols, aromatics, esters, ketones, and furanone decreased their percentage with roasting intensity. These groups are related to the positive aroma. As the roasting time increased, the smell of burning appeared; hence, the previous groups lost their positive aroma [36,37]. Finally, lactones, pyrroles, and aldehydes appeared in all the samples and did not present important differences between the different qualities analyzed.

The main VOCs identified and their percentage content in the odor are shown in Table 4. The 10 most representative volatile compounds in the samples were determined. The compounds 2-methyl-furan, 2,5-dimethyl-pyridine, 2-methyl-butanal, and 2-furfurylthiol increased in proportion from t6 to t11, reaching a maximum at t11. These compounds have a negative roasted aroma, and the above VOCs are related to the poor quality of roasted coffee [15,38]. On the contrary, there were other VOCs that appeared in greater proportions in t6 and decreased their contents with increasing thermal intensities. Compounds such as 1-(4-nitrophenyl)-3-phenylamino-propenone and nonanoic acids decreased their contents during thermal treatment, whereas 2-methoxy-4-vinylphenol and 2-phenylethyl alcohol had the highest proportions at t6 and with the highest thermal intensity, these aromas decreased, as has been previously reported [39]. All these compounds are related to the positive aroma [9]. The thermal intensity did not affect 2(5H)-furanone and 1-methyl pyrrole. These VOCs have a buttery aroma and coffee aroma [40].

### 3.3. Relationship between the Aromatic Profile and E-Nose of Roasted Coffee Beans

**Figure 7:**
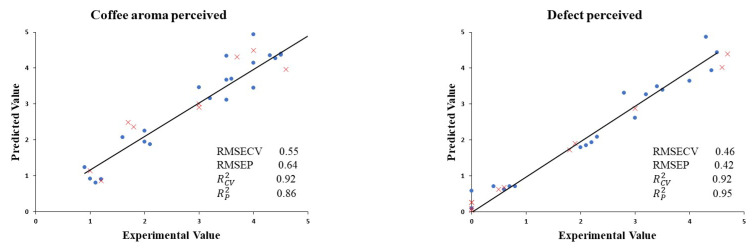
Experimental values against PLS cross-validation predictions (●) and validation set predictions (x) for coffee bean aromas and defect aromas perceived by panelists.

Once the sensory profile of the coffee samples subjected to different thermal treatments was known (Table 3), a relationship between the positive and negative aromas perceived by the tasters and the data obtained by the E-nose was established. Thus, with the aim of assessing the use of the electronic devices to quantify the quality parameters in roasted coffee beans, a partial least squares (PLS) chemometric approach was conducted (Figure 7) with data obtained by the E-nose and those obtained by sensory analyses previously conducted. PLS regression was used to establish prediction models from the coffee aroma perceived by the tasting panel and the data produced by the E-nose signals. $R_{CV}^{2}$ values for the models established for fruity and coffee aromas were 0.92. Low RMSECV values of 0.55 were also estimated. The PLS calibration model was validated using samples that were not included in the calibration test. The validation parameters obtained were also acceptable; $R_{P}^{2}$ values were 0.86 for the coffee aroma perceived, whilst RMSEP values were 0.64. At the same time, PLS regression was conducted to set a prediction model from the defects perceived by the tasting panel and the data produced by the E-nose signals. $R_{CV}^{2}$ values for the models established for the defects perceived were 0.92. These defects were related to the aroma of the roast intensity. Low RMSECV values of 0.46 were also estimated. The PLS calibration model was also validated using samples that were not included in the calibration test. The validation parameters obtained were also acceptable. $R_{P}^{2\;}$ values were 0.95 for the coffee aroma perceived, whilst RMSEP values were 0.42. Thus, this model allows for the quantification of coffee aromas of roasted samples using the E-nose.

Several authors have determined the quality of Robusta coffee submitted to different roasting degrees by using electronic noses and chemometric multivariate analyses [41]. Results are similar to those observed in the present study, being able to establish a quantitative model between sensor responses and the physicochemical parameters of the roasted coffee. Zhang et al. [34] indicated the aroma profile of Robusta coffee beans by using chromatography–mass spectrometry and electronic nose analysis during the drying process of the product, providing theoretical evidence about the change rule in the aromas of coffee beans during drying processes. According to these data, Bona et al. [42] suggested that the future of this electronic device seems promising because researchers are increasing their attempts to develop innovative instrumental techniques and pattern recognition tools. Results obtained in this work demonstrate that the sensors of the designed electronic device are capable of discriminating Arabica coffee samples based on their sensory qualities during different elaboration processes. This chemometric tool would be useful for the industry since the E-nose allows for a quick and efficient preliminary classification of the quality of coffee beans before they are subjected to a subsequent roasting process. This would allow for a quality classification of roasted coffee to be made from the first phase of coffee processing.

## 4. Conclusions

The electronic device was able to discriminate between roasted coffee beans subjected to different thermal treatments. The electronic sensors reacted with different intensities depending on the sample studied. PLS-DA is a useful model that can predict correctly the roasted coffee beans analysed. The differences detected by the E-nose are due to the fact that the samples studied presented positive and negative aromas, confirmed by the tasting panel and the identification of VOCs. The compounds 2-methyl-furan, 2,5-dimethyl-pyridine, 2-methyl-butanal, and 2-furfurylthiol are related to negative aromas in coffee and have a differentiating effect in the samples. Finally, the use of electronic devices can be valid to quantify the quality parameters in roasted coffee beans by using the PLS chemometric approach. According to Sánchez et al. [28], the E-nose is a rapid, inexpensive, nondestructive, and environmentally friendly qualitative analysis tool that can be used for classifying coffee qualities during the roasting process. Finally, it should be noted that one of the current limitations in E-nose development is the inherent drift of the gas sensors, which result in a random temporal variation of the sensor responses when exposed to the same scents under identical conditions. This causes the patterns previously learned to generate the model to become obsolete after a short time, losing the electronic system’s ability to identify known odors. It would be necessary to develop in future works protocols and mathematical models that correct this drift.

## Figures and Tables

**Table 1:** Description of the 11 signals generated by the E-nose device.

Codes	Signal	Sensor
T	Temperature (°C)	BME680
P	Pressure (hPa)	BME680
H	Humidity (%RH)	BME680
1	Gas Measurement (Ω)	BME680
2	_e_CO_2_ (ppm)	SGP30
3	TVOC (ppb)	SGP30
4	H_2_	SGP30
5	Ethanol	SGP30
6	_e_CO_2_ (ppm)	CCS811
7	TVOC (ppb)	CCS811
8	Sensor Resistance (Ω)	CCS811
9	_e_CO_2_ (ppm)	iAQ-Core
10	TVOC (ppb)	iAQ-Core
11	Sensor Resistance (Ω)	iAQ-Core

**Table 2:** Confusion matrix obtained through PLS-DA for roasted coffee beans submitted to different thermal treatments (t6–t11). Values are expressed in percentages of the samples studied.

	Predicted Class					
Real Class	t6	t7	t8	t9	t10	t11
t6	16.6	0	0	0	0	0
t7	0	16.6	2.7	0	0	0
t8	0	0	13.8	2.7	0	0
t9	0	0	0	13.8	0	0
t10	0	0	0	0	16.6	0
t11	0	0	0	0	0	16.6

**Table 3:** Positive (coffee) and negative aromas (roasted/burnt) for roasted coffee beans submitted to different thermal treatments (t6–t11). Different lowercase letters mean statistically significant differences between samples submitted to different thermal treatments in each sensory attribute (one-way ANOVA followed by Tukey’s test, *p* < 0.05). n.d., not detected.

t (min)	Aroma	
	Coffee	Roasted/Burnt
t6	1.1 ± 0.1 a	n.d.
t7	2.7 ± 0.2 b	n.d.
t8	3.8 ± 0.2 b	n.d.
t9	5.7 ± 0.3 b	2.1 ± 0.2 a
t10	4.4 ± 0.2 b	3.2 ± 0.3 a
t11	3.2 ± 0.2 b	4.4 ± 0.2 a

**Table 4:** Relative percentage of volatile compounds (VOCs) obtained for roasted coffee beans submitted to different thermal treatments (t6–t11).

CAS Number	VOC’s	t6	t7	t8	t9	t10	t11
98 497-23-4	2(5H)-furanone	1.9	2.8	2.4	2.8	1.8	1.6
3777-69-3	2-Methyl-furan	3.5	9.8	12.1	11.2	19.8	20.4
24683-00-9	2,5-Dimethyl-pyridine	8.3	10.2	14.6	17.8	19.8	25.6
96-54-8	1-Methyl pyrrole	2.9	2.4	2.4	2.3	2.2	1.8
96-17-3	2-Methyl-butanal	4.4	9.1	12.1	14.0	19.8	18.8
1000302-96-9	1-(4-Nitrophenyl)-3-phenylamino-propenone	10.1	10.2	9.7	7.0	0.0	0.0
112-05-0	Nonanoic acid	8.2	11.8	14.6	14.0	4.3	2.6
7786-61-0	2-Methoxy-4-vinylphenol	27.7	19.7	10.7	9.3	7.2	0.0
60-12-8	2-Phenylethyl alcohol	29.7	17.3	10.7	9.8	11.5	5.7
98-02-2	2-Furfurylthiol	3.3	6.7	10.7	11.7	13.7	23.5

## Data Availability

All relevant data are included within the manuscript. The raw data are available on request from the authors.

## Sample Availability

Samples of the study are available from the authors.
